# Transcriptome-Based Identification of Reference Genes for Expression Analysis in Cassava Under *Xanthomonas phaseoli* pv. *manihotis* Infection

**DOI:** 10.3390/plants14233655

**Published:** 2025-11-30

**Authors:** Jing Yang, Ciyun Li, Jie Chen, Dongying Lu, Qi Yang, Ruotong Li, Liyun Yang, Xiaofei Zhang, Yinhua Chen, Shousong Zhu, Xiaolei Niu

**Affiliations:** 1 School of Breeding and Multiplication (Sanya Institute of Breeding and Multiplication), School of Tropical Agriculture and Forestry, Hainan University, Sanya 572025, China; 15619154888@163.com (J.Y.); lcylcy6400@163.com (C.L.); 20203104280.hainanu@vip.163.com (J.C.); ludongyingi@163.com (D.L.); wkjb24@163.com (Q.Y.); liruotong1220@163.com (R.L.); 996674@hainanu.edu.cn (L.Y.); yhchen@hainanu.edu.cn (Y.C.); 2 National Key Laboratory for Tropical Crop Breeding, Sanya 572025, China; 3 CGIAR Research Program on Roots Tubers and Bananas (RTB), International Center for Tropical Agriculture (CIAT), Cali 763573, Colombia; xfczhang@ucdavis.edu

**Keywords:** *Manihot esculenta* Crantz, RT-qPCR, biotic stress

## Abstract

Reverse transcription quantitative PCR (RT-qPCR) is a powerful and widely used technique for quantifying alterations in gene expression. Cassava bacterial blight caused by *Xanthomonas phaseoli* pv. *manihotis* severely constraints cassava growth and yield. Accurate evaluation of the expression levels of genes following infection by *X. phaseoli* pv. *manihotis* is crucial for the identification of potential cassava resistance genes. In this study, thirty-two novel potential reference genes were screened from the cassava–*X. phaseoli* pv. *manihotis* transcriptome. Their expression, along with that of seven literature-reported cassava reference genes, was evaluated in two susceptible and two resistant cassava varieties at six time points post-inoculation by *X. phaseoli* pv. *manihotis* through RT-qPCR analysis. The stability of thirty-nine candidate reference genes was assessed by four algorithms: geNorm, NormFinder, Delta Ct, and RefFinder. The results demonstrated that serving as new reference genes, *MehnRNPR* and *MePRPF38B* consistently exhibited superior expression stability over seven established reference genes under *X. phaseoli* pv. *manihotis* infection, regardless of the susceptible or resistant cassava varieties. The reliability of the reference genes was validated by assessing the expression pattern of *MeNAC35* and *MeSWEET10a* under *X. phaseoli* pv. *manihotis* infection. The findings of this study provide valuable insights for advancing the precision of the quantification of cassava candidate genes associated with disease resistance.

## 1. Introduction

Cassava (*Manihot esculenta* Crantz) is a perennial vegetatively propagated shrub widely grown in tropical and subtropical regions for starchy tuberous roots. Cassava is a vital crop for food security, serving as a dietary staple food for over one billion people worldwide [1,2]. Additionally, cassava starch is used as livestock feed and as an important raw material for various industrial applications including bioethanol production, textile processing, and pharmaceutical manufacturing [3]. However, cassava bacterial blight (CBB), caused by *Xanthomonas phaseoli* pv. *manihotis*, is considered as the most devastating bacterial disease of cassava, resulting in yield losses of up to 100% under favorable climatic conditions [4]. CBB has been reported in all regions where cassava is cultivated, posing a critical threat to food security and economic viability of cassava production. As effective chemical control methods for CBB are still unavailable, the priority approach for CBB management is to breed cultivars with high levels of resistance [5]. Therefore, the analysis of gene expressions responsive to *X. phaseoli* pv. *manihotis* infection and the identification of cassava resistance genes at the molecular level are of crucial importance, as they provide valuable genetic resources for developing cassava cultivars with enhanced CBB resistance.

Techniques for gene expression analysis in biological research mainly include semiquantitative reverse transcription, Northern blot, in situ hybridization, gene chips, RNA sequencing, and reverse-transcription quantitative PCR (RT-qPCR). Among these, RT-qPCR is widely regarded as the favored technique for the detection of gene expression due to its high sensitivity, specificity, reproducibility, and high throughput. However, the accuracy and reliability of RT-qPCR can be profoundly impacted by various factors, such as RNA integrity and amount, reverse transcription efficiency, initial template input, qPCR amplification efficiency, and inherent technical variations [6]. To correct for these variables and ensure accurate measurements, the use of stably expressed reference genes as an internal control is a prerequisite. Ideal reference genes should maintain stable expression across the samples to be compared. Using inappropriate reference genes can compromise RT-qPCR precision or even result in incorrect conclusions [7,8].

In the pre-genomic era, reference genes were selected as internal controls mainly involved in basic cellular processes, such as actin (*ACT*), tubulin (*TUB*), 18s rRNA, elongation factor 1-α (*EF1α*), and polyubiquitin (*UBQ*), and these genes were assumed to be constantly expressed. Unfortunately, numerous studies have shown that the traditional reference genes varied largely in diverse plant species under various experimental conditions. Recent advancements in high-throughput sequencing technologies, particularly in microarray gene chips and transcriptome profiling, have offered invaluable resources for screening novel reference genes. The novel reference genes selected from transcriptome sequencing data have shown superior stability compared to those traditionally used ones in a range of plant species, including *Arabidopsis* [9], barley [10], wheat [11,12], rice [13,14], buckwheat [15], grape [16], maize [17], soybean [18], tomato [19,20], cotton [21], polygonaceae [22], dayflower [23], taro [24], and bamboo [25]. However, in contrast to extensive studies on reference gene validation in plant cultivars, tissues, developmental stages, and abiotic conditions, only limited research has been conducted under plant biotic stress conditions. In plant pathosystems, diverse pathogenic microbes including viruses, fungi, and bacteria can induce metabolic alterations and gene-expression reprograming in host plants [26,27,28,29]. Thus, identifying reliable reference genes from transcriptomic profiling under pathogen infection is critical for the investigation of molecular mechanisms related to disease and discovery of host disease-resistant genes. For example, in grape infected by leafroll-associated virus 3, the stable reference genes were selected as *CYSP*, *NDUFS8*, and *YSL8* [30]; when infected with gray mold, *VIT-17s0000g02750* and *VIT-06s0004g04280* exhibited the most stable expression [31]. Similar results were obtained in tomato leaves infected with *Begomovirus* [32], *Pseudomonas* [33], *Ralstonia* [34], and *Xanthomonas* [35], respectively.

However, to date, no studies have used genome-wide screening to identify reference genes with stable expression in cassava–*X. phaseoli* pv. *manihotis* pathosystem. *X. phaseoli* pv. *manihotis* HN01, a highly virulent strain of *X. phaseoli* pv. *manihotis,* has emerged as a predominant pathogenic bacterium constraining cassava yield across all cassava growing regions in China [36]. In this study, based on the transcriptomic sequencing data in cassava, thirty-two genes were screened as prospective candidate reference genes, in response to *X. phaseoli* pv. *manihotis* CHN01 infection. Their expression stability, along with that of seven literature-reported reference genes, was assessed in two susceptible and two resistant cassava varieties at six time points post-inoculation by *X. phaseoli* pv. *manihotis* using RT-qPCR. Subsequently, four statistical algorithms, geNorm [37], NormFinder [38], Delta Ct [39], and RefFinder [40], were employed to evaluate the expression consistency of the candidate genes. In addition, *MeNAC35* and *MeSWEET10a*, involved in cassava responses under *X. phaseoli* pv. *manihotis* infection [41,42], were tested for validation. The findings of this study contribute appropriate reference genes for future studies on excavating disease-resistant genes and exploration of disease-resistant mechanisms on the molecular level in cassava–*X. phaseoli* pv. *manihotis* pathosystem.

## 2. Results

### 2.1. Confirmation of X. phaseoli pv. manihotis Infection and Sample Conditions

**Figure 1:**
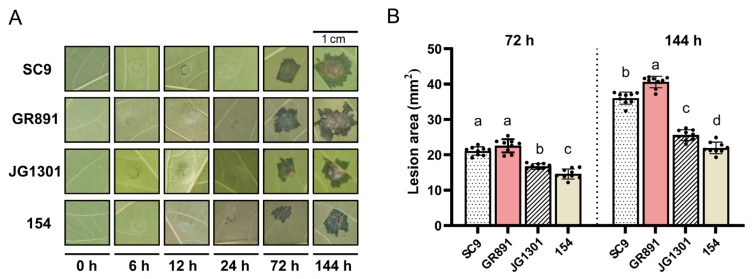
Symptoms of four cassava varieties induced by *X. phaseoli* pv. *manihotis* CHN01 infection (**A**) and lesion areas of four cassava varieties (**B**) at different time points post-inoculation. Different letters a, b, c, d above the bars indicate lesion areas that are significantly different (*p* < 0.05) from each other as determined by one-way ANOVA (SPSS V20.0) using the Tukey–HSD method. Error bars represent standard deviation.

Fully expanded mature leaves of susceptible and resistant cassava varieties were inoculated with *X. phaseoli* pv. *manihotis* CHN01, respectively. As shown in Figure 1, typical water-soaking symptoms in *X. phaseoli* pv. *manihotis*-infected leaves were observed at 72 h post-inoculation (hpi), and progressed at 144 hpi. The lesion areas in two susceptible cassava varieties, GR891 and SC9, were significantly bigger than those in the resistant cassava varieties, JG1301 and 154 (an offspring of GR891-selfing progeny), both at 72 and 144 hpi. Among the four tested varieties, GR891 exhibited the largest lesion areas, followed by SC9, JG131, and 154 at 144 hpi. Between the two susceptible cassava varieties, GR891 exhibits more susceptibility than SC9, whereas between the two resistant varieties, 154 shows stronger resistance than JG1301 at 144 hpi. Leaves from four cassava varieties at six time points post *X. phaseoli* pv. *manihotis* inoculation were collected for RNA isolation and subsequent RT-qPCR analysis.

### 2.2. Reference Gene Selection, Primer Specificity, and PCR Efficiency

Based on the selection criteria derived from cassava–*X. phaseoli* pv. *manihotis* RNA-seq data, thirty-two reference genes were chosen as potential reference genes. Additionally, seven reference genes obtained from published literature were included. In total, thirty-nine genes were selected for further analysis to access their stability profiles following *X. phaseoli* pv. *manihotis* CHN01 infection, as described above. The gene name, identifier, product size, correlation coefficient (R^2^), amplification efficiency, and description are shown in Table 1. The primer sequences of all genes are provided in Table S1. The specificity of the primers was determined by RT-qPCR melting curve analysis. All primers for the candidate reference genes displayed a single peak, indicating that the primers had satisfactory specificity (Figure S1). The amplification product lengths ranged from 106 to 210 bp (Table 1). The amplification efficiency (E) of all 39 reference gene reactions varied from 90.7% for *MePLAC8* to 109% for *MeTIA1*, which were all within the acceptable range of 90–110% (Table 1). Furthermore, correlation coefficients (R^2^) ranged from 0.982 to 0.999 (Table 1). The above results indicated that the primer pairs of these 39 candidate reference genes were suitable for subsequent RT-qPCR experiments.

### 2.3. Expression Analysis of the 39 Reference Genes Under X. phaseoli pv. manihotis Infection by RT-qPCR

**Figure 2:**
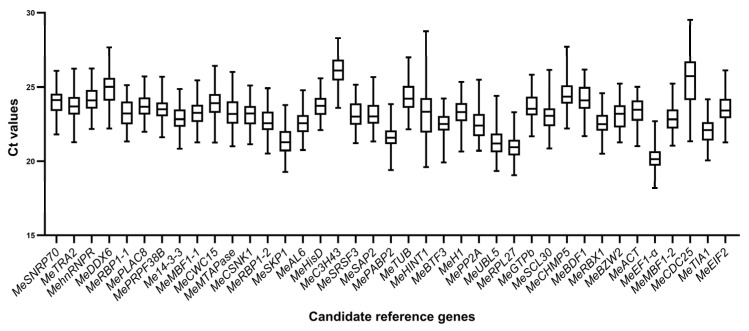
Ct values of the 39 candidate reference genes in cassava–*X. phaseoli* pv. *manihotis* interaction. The line across the box displays the median values. Lower and upper boxes represent the 25th percentile to the 75th percentile. Whiskers indicate the maximum and minimum Ct values.

The expression levels of the 39 candidate reference genes were evaluated in 72 samples collected from leaves of four cassava varieties infected with *X. phaseoli* pv. *manihotis* CHN01 at six time points (three biological replicates per each time point), as mentioned above. The transcriptional abundances were measured by calculating the RT-qPCR Ct value (Table S2). Ct values directly reflect the abundance of gene expression, with smaller Ct values indicating higher levels of gene expression. The Ct values for all 39 potential reference genes in all samples are plotted in Figure 2. Across all samples, the Ct values of the genes exhibited a distribution between 18.19 and 29.52 for *MeEF1α* and *MeCDC25*, respectively. *MeEF1α* exhibited the highest expression level among 39 reference genes, characterized by the lowest mean Ct value (20.20), followed by *MeRPL27* (20.94). In contrast, *MeC3H43* had the highest average Ct values with the lowest expression levels (26.07). The standard deviation (SD) of the Ct values represented the variation in gene expression across the samples. *MeCDC25* and *MeHINT1* displayed the largest variation in expression levels, with SD values of 2.01 and 1.80, respectively, whereas *MeHisD* and *MePRPF38B* exhibited the lowest variability, with SD values of 0.73 and 0.77. Nevertheless, to guarantee the accuracy of reference gene evaluation, their stability was assessed using the following four algorithms.

### 2.4. Stability Analysis of the 39 Selected Reference Genes Using Different Algorithms

To further evaluate the consistency in expression of the 39 candidate reference genes, four different algorithms (geNorm, NormFinder, Delta CT, and RefFinder) were used to assess their stability under *X. phaseoli* pv. *manihotis* CHN01 infection at six time points. The samples were categorized into five groups: susceptible variety SC9, susceptible variety GR891, resistant variety GR891, resistant line 154, and the overall dataset (all samples).

#### 2.4.1. GeNorm Analysis

The GeNorm algorithm assessed the stability of reference genes by calculating the gene-stability measure value (M). A smaller M value indicates a higher stability in gene expression. Additionally, a gene possessing an M value below 1.5 is considered suitable as a reference gene [37]. Across the six time points assessed (0, 12, 24, 48, 72, and 144 h post-inoculation with *X. phaseoli* pv. *manihotis*), all candidate genes showed expression stability lower than 0.9 in all five groups, indicating that all reference genes were suitable, as shown in Table 2. The stability of the candidate reference genes varied among the five groups. Under *X. phaseoli* pv. *manihotis* inoculation, in cassava susceptible variety SC9 and resistant variety JG1301, *MehnRNPR* had the lowest M value and was regarded as the most stable reference gene. However, in cassava susceptible variety GR891 and resistant line 154, *MeSKP1* and *MeEIF2* emerged as the most consistent reference genes. For all samples, *MehnRNPR* and *MeRBP1-2* ranked as the most stable pair of reference genes, followed by *MeAL6,* whereas *MeCDC25* was the least stable gene. Interestingly, *MePP2A* and *MeTUB*, two literature-reported reference genes, were identified as the second unstable reference genes among the 39 reference genes in susceptible varieties SC9 and GR891 under *X. phaseoli* pv. *manihotis* infection, respectively. In addition, the geNorm algorithm determines the optical number of reference genes by calculating pairwise variation (Vn/n + 1). When Vn/n + 1 is below the cutoff of 0.15, the optimal number of reference genes is n. In this study, calculated pairwise variations of all five groups were less than 0.15, which suggested the two reference genes were adequate for normalizing RT-qPCR results (Table S3).

#### 2.4.2. NormFinder Analysis

The NormFinder algorithm evaluated the expression stability of candidate reference genes based on intra- and inter-group expression variations, and ranked them according to their stability value (S) [38]. A lower stability value indicates more stable gene expression. According to the stability value calculated by the NormFinder algorithm across the six time points post-inoculation by *X. phaseoli* pv. *manihotis*, *MehnRNPR* was identified as the most reliable reference gene across all five groups, with the exception of the SC9 variety. In contrast, *MeCDC25* showed the lowest stability (Table 3). Meanwhile, *MePRPF38B* emerged as the most consistent gene in SC9 and ranked second and third in GR891 and JG1301, respectively, following *X. phaseoli* pv. *manihotis* infection (Table 2). Again, *MePP2A* and *MeTUB* were identified as the second-most unstable reference genes in susceptible varieties SC9 and GR891 under *X. phaseoli* pv. *manihotis* infection, respectively.

#### 2.4.3. Delta Ct Analysis

The Delta Ct algorithm analyzed the stability of the candidate reference genes by comparing the relative expression of gene pairs within each sample, using the mean standard deviation (mSD) value. A lower mSD value suggested high stability. In susceptible cassava varieties SC9 and GR891, *MePRPF38B* was shown to be the most stable reference gene, whereas in resistant cassava variety JG1301 and line 154, the most stable reference gene was *MehnRNPR.* The second stable reference gene analyzed by the Delta Ct algorithm varied among the four cassava varieties, as shown by *MehnRNPR* in SC9 and GR891, *MeRBX1* in JG1301, and *MePRPF38B* in 154, respectively. *MehnRNPR* and *MePRPF38B* were shown to be the most stable genes in all samples, followed by *MeRBP1-2, MeRBX1*, and *MeAL6*, whereas *MeCDC25* was the least stable (Table 4). In line with the results of geNorm and NormFinder, *MePP2A* and *MeTUB* ranked as the second-most unstable reference genes in susceptible varieties SC9 and GR891 under *X. phaseoli* pv. *manihotis* infection, respectively (Table 4).

#### 2.4.4. RefFinder Analysis

To avoid the discrepancies evaluated by a single algorithm for stability, we utilized the online RefFinder tool to comprehensively assess the stability of the candidate reference genes by calculating the geomean of ranking values [40]. A lower geometric mean (GeoM) indicates a higher gene expression stability. As shown in Table 5, *MeAL6* and *MePRPF38B* were the most stable reference genes in susceptible cassava varieties SC9 and GR891, respectively, whereas *MehnRNPR* ranked at the top in the resistant cassava variety JG1301 and line 154 across all *X. phaseoli* pv. *manihotis* inoculation stages. For all samples, *MehnRNPR* was the optimal candidate reference gene, followed by *MePRPF38B* and *MeRBP1-2. MeCDC25* was the most unstable reference gene in all groups except in susceptible cassava susceptible variety SC9. In addition, *MePP2A* and *MeHINT1* were suggested to be the most unsuitable reference genes in SC9 variety. *MeTUB* ranked as the second- and third-most unstable reference gene in cassava varieties GR891 and JG1301, respectively. In general, among four algorithms, *MehnRNPR*, *MePRPF38B*, *MeRBP1-2*, *MeRBX1*, and *MeAL6* appeared more times at the front of the ranking, indicating these five genes are more stable. In contrast, *MeCDC25* and *MeHINT1* were the least stable reference genes.

### 2.5. Validation of Candidate Reference Genes

**Figure 3:**
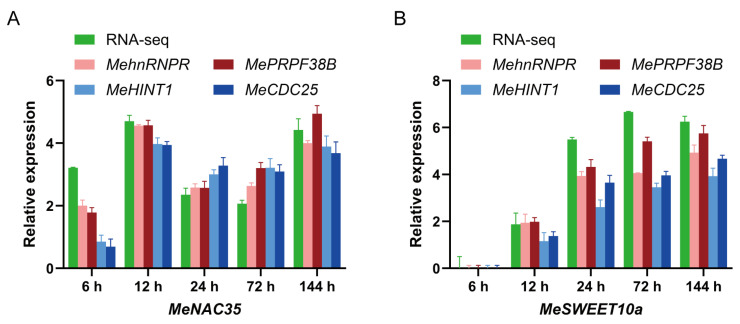
The relative expression of *MeNAC35* (**A**) and *MeSWEET10a* (**B**) were determined in cassava leaves at different time points upon *Xpm* infection by the use of select reference genes, including the most or least stable reference genes for normalization. The units of RNA-seq data are multiples of log2 (fold change). Error bar represents standard deviation.

Based on the transcriptomic data of cassava–*X. phaseoli* pv. *manihotis* interaction (PRJNA881631), it was identified that *MeNAC35* (Manes.03G114200) and *MeSWEET10a* (Manes.06G123400) were significantly differentially expressed in response to *X. phaseoli* pv. *manihotis* infection at six time points post-inoculation [41,42]. To validate the reliability of the chosen stable reference genes, the relative expression pattern of *MeNAC35* and *MeSWEET10a* was assessed using the top-two and bottom-two performing reference genes in cassava SC8 variety under *X. phaseoli* pv. *manihotis* infection. The top-two performing reference genes, *MehnRNPR* and *MePRPF38B*, together with the bottom-two reference genes, *MeHINT1* and *MeCDC25*, were employed for RT-qPCR evaluation and then compared with the transcriptome data. When the most stable genes of *MehnRNPR* and *MePRPF38B* were used for normalization, the relative expression trends of *MeNAC35* and *MeSWEET10a* were similar to the transcriptome data (Figure 3A). In contrast, when the two least stable reference genes were used as the reference gene, the expression trends of *MeNAC35* and *MeSWEET10a* varied greatly compared to the transcriptome data (Figure 3B). These findings demonstrated that the chosen reference genes were validated as accurate and reliable.

## 3. Discussion

Cassava bacterial blight is a serious bacterial disease caused by *X. phaseoli* pv. *manihotis*, which dramatically dampened the growth and production of cassava [4]. Breeding disease-resistant cassava varieties is the most effective approach to managing CBB [5]. Expression profiling of gene changes in cassava–*X. phaseoli* pv. *manihotis* interaction provides a valuable resource for excavating the cassava resistance genes and elucidating the resistance mechanism at the molecular level [47]. Transcriptome analysis has become a powerful technology used for gene expression studies in various organisms and different treatments [48]. The data generated by RNA-seq have been extensively used in the plant research field for the selection of novel candidate reference genes [14,23,24].

In the previous studies, Hu et al. identified twenty-six cassava reference genes through analyzing their thirty-two cassava transcriptome data, and verified that *MeBTF3*, *MeHisD*, and *MeC3H43* were the best reference genes in six cassava varieties (Arg7, KU50, W14, SC124, SC5, and Rongyong9) under different developmental and environmental conditions [43]. Moreno et al. found *MePP2A* and *MeGTPb* were the most stable reference genes in three CBSV-infected cassava varieties [45]. In addition, *MePP2A*, *MeGTPb*, *MeTUB*, and *MeEF1α* were used as reference genes for the normalization of gene expression in cassava upon *X. phaseoli* pv. *manihotis* infection [41,44,46]. However, there are no reports on the identification of stable reference genes for normalizing gene expression in cassava upon *X. phaseoli* pv. *manihotis* infection of susceptible and resistant genotypes. In this study, based on the cassava–*X. phaseoli* pv. *manihotis* transcriptome datasets, we identified and validated thirty-two novel candidate cassava reference genes with superior expression stability challenged by the bacterial pathogen *X. phaseoli* pv. *manihotis*, and compared them with seven literature-reported reference genes. The screening method was similar to the approach demonstrated in previous studies [24,31,43,49]. With the specific screening criteria, our novelly selected thirty-two candidate reference genes mainly function in fundamental cellular processes including RNA binding, RNA processing, RNA transcription, cell cycle control, chromatin modification, protein synthesis, and protein degradation.

Four different algorithms, geNorm, NormFinder, Delta-CT, and RefFinder, were adopted to evaluate the stability of gene expression in cassava under *X. phaseoli* pv. *manihotis* infection. In the analysis using the geNorm algorithm, the stability value of all thirty-nine genes was below the threshold of 1.5, indicating that all selected reference genes were suitable. Relatively, *MeCDC25* and *MeHINT1* had the worst stability among the 39 candidate reference genes analyzed by the four algorithms. This indicates that *MeCDC25* and *MeHINT1* are not suitable as an internal reference gene for cassava infection by *X. phaseoli* pv. *manihotis*. Additionally, in the geNorm analysis, all pairwise-variation values were below 0.15, suggesting no need to introduce a third reference gene for normalization. GeNorm analysis showed that *MehnRNPR* performed best in SC9, JG1301, and the entire dataset (all samples), whereas *MeSKP1* and *MeEIF2* ranked first in GR891 and 154. Conversely, *MePRPF38B* displayed higher stability in SC9 by NormFinder and Delta CT analyses. GeNorm, NormFinder, and Delta CT algorithms showed some discrepancies in the ranking of the 39 candidate reference genes, which was also observed in the previous studies and is probably due to differences in these mathematical models [25,31]. RefFinder was therefore used to comprehensively assess the expression stability of 39 candidate reference genes. Based on the results of RefFinder, *MehnRNPR* and *MePRPF38B* were recommended as the most stable reference genes in all groups except in SC9. Interestingly, *MeAL6* ranked first in SC9 by RefFinder, followed by *MePRPF38B* and *MehnRNPR*.

MehnRNPR and MePRPF38B are involved in RNA processing, and MeAL6 is a PHD finger protein, which participates in chromatin modification. In line with these results, based on microarray analyses, tomato *PHD* and RNA-processing *LSM7* genes were identified and validated as the most stably expressed reference genes in *Xanthomonas campestris* pv. *vesicatoria*-infected tomato leaves [35]. *MeBTF3*, *MeHisD*, and *MeC3H43*, which showed stable expression in the cassava developmental stage, were not the optimal reference genes upon *X. phaseoli* pv. *manihotis* infection in this study. Interestingly, the commonly used five reference genes in the cassava–*X. phaseoli* pv. *manihotis* pathosystem, *MeACT*, *MeEF1a*, *MeTUB*, *MeGTPb*, and *MePP2A*, all turned out to be unstably expressed. Similarly, in the *X. campestris* pv. *vesicatoria*–tomato pathosystem, tomato *ACT*, *TUB*, and *EF-1α* were identified as unstable reference genes [35]. In tomato leaf interactions with *Pseudomonas fluorescens* 55, *EF1α* was included within the least stable expression group [33]. In *Nicotiana benthamiana* leaves infiltrated with *P.fluorescens* 55, *NbPP2a* and *NbEF1α* exhibited variable gene expression [50]. In addition, in kiwifruit leaves infected with *Pseudomonas syringae* pv. *actinidiae*, *ACT*, *TUB*, and *EF-1α* were marked as the unstable reference genes, whereas *PP2A* was the most stable gene [51]. In cucumber infected with *Pectobacterium brasiliens*, *CsACT*, *CsEF-1α*, and *CsPP2A* were not the optimal reference genes [52]. Contrastingly, in potato infected by *Pectobacterium atrosepticum*, *ACT* and *EF1α* exhibited the highest stability [53]. *GTPb* was the most appropriate reference genes in wheat and cassava infected with different viruses [41,54]. These findings highlight that suitable reference genes need to be identified and tested for each pathogenic Gram-negative bacterium–host pathosystem.

To confirm the reliability of the proposed reference genes, two genes (*MeNAC35* and *MeSWEET10a*) from the cassava–*X. phaseoli* pv. *manihotis* transcriptome data were selected and assessed for their relative expression pattern using the two most and two least stable reference genes identified under *X. phaseoli* pv. *manihotis* infection. When *MeHINT1* and *MeCDC35* were used as reference genes, the expression patten of these two genes varied compared to the transcriptome data. However, when employing *MehnRNPR* and *MePRPF38B* for normalization, the expression patterns of these two genes closely paralleled the expression pattern observed in the transcriptome data. These results highlight the critical role of using stable reference genes for investigating the expression of pivotal genes in the cassava–*X. phaseoli* pv. *manihotis* pathosystem.

In summary, in this study, the expression stability of 39 candidate reference genes was investigated using four statistical algorithms across two resistant and two susceptible cassava varieties inoculated with *X. phaseoli* pv. *manihotis*, with sampling conducted at six distinct time points post-inoculation. In addition, the relative expression patterns of *MeNAC35* and *MeSWEET10a* in cassava leaves infected by *X. phaseoli* pv. *manihotis* were analyzed to validate the reliability of the identified stable reference genes. Through screening and validation, the *MehnRNPR* and *MePRPF38B* genes were identified as the stable reference genes in the cassava–*Xpm* pathosystem. This is the first comprehensive study to identify and validate reference genes with RT-qPCR analysis in cassava during *X. phaseoli* pv. *manihotis* infection, encompassing both susceptible and resistant genotypes. The results will facilitate understanding the regulatory networks of host–pathogen interactions and further contribute to the identification of genes related to CBB resistance and prioritize the most promising genes for functional validation and marker-assisted cassava breeding selection.

## 4. Materials and Methods

### 4.1. Plant Material and X. phaseoli pv. manihotis CHN01 Inoculation

The cassava susceptible varieties SC9, GR891, and SC8 (for validation) and resistant varieties JG1301 and line 154 (an offspring of GR891-selfing progeny) were used in this study [55]. Stems of four cassava varieties, approximately 15 cm in length with two to three buds, were sub-cultured in plastic pots containing an equal mix of vermiculite and nutrient soil in a greenhouse with a 12 h/12 h photoperiod at 28 °C, 60–70% relative humidity, and with a light intensity of 130–150 µmol/m^−2^ s^−1^. The pathogenic bacterium *X. phaseoli* pv. *manihotis* CHN01 strain was cultured in solid LPGA medium at 28 °C and diluted to OD_600_ ≈ 0.1, approximately 10^8^ CFU ml^−1^ using 10 mM MgCl_2_. Three expanded leaves from uniformly growing plants were infiltrated with *X. phaseoli* pv. *manihotis* CHN01 using a 1 mL sterile needleless syringe. Disease symptoms of infected cassava leaves were photographed at the six time points post-inoculation, and nine lesion areas were calculated by Image J software (V1.8.0) and analyzed by SPSS software (V20.0). Infected leaves were collected at six time points post-inoculation: 0 h post-inoculation (hpi), 6 hpi, 12 hpi, 24 hpi, 72 hpi, and 144 hpi. Leaves collected at time 0 h, immediately after infiltration, served as the control. All harvested leaves were immediately frozen in liquid nitrogen and stored at −80 °C for subsequent RNA isolation. All experiments were performed in triplicate and repeated at least three times.

### 4.2. Total RNA Extraction and cDNA Synthesis

Total RNA was extracted from cassava leaves using an RNAprep Pure Plant Plus Kit (TIANGEN, Beijing, China), according to the manufacturer’s instructions. RNA concentration and purity were analyzed using a Nanodrop 2000 spectrophotometer (Thermo, Wilmington, DE, USA). RNA integrity was determined by 1.0% agarose gel electrophoresis. Only RNA samples exhibiting an A260/A280 ratio ranging from 1.8 to 2.1 and an A260/A230 ratio above 1.8 were selected for subsequent cDNA synthesis. For each sample, 1 ug of total RNA was reverse-transcribed to cDNA using SPARKscript II All-In-One RT Super Mix (with gDNA eraser) (Spark, Shandong, China) according to the manufacturer’s protocol. Three biological replicates were included for each time point of infected leaves for RNA isolation and cDNA synthesis. The synthesized cDNAs were stored at −20 °C until further use.

### 4.3. Selection of Candidate Reference Genes and Primer Design

The transcriptome data of cassava SC9 leaves infected with *X. phaseoli* pv. *manihotis* were obtained from the NCBI database under accession number PRJNA881631 [56]. Additionally, novel transcriptome sequencing of leaves from different cassava genotypes infected with *X. phaseoli* pv. *manihotis* at different time points post-inoculation were performed using the Illumina NovoSeq 6000 platform, and the raw sequencing data were deposited in the China National Center for Bioinformation database under accession number PRJCA049847. Briefly, total RNA was extracted from *X. phaseoli* pv. *manihotis*-infected cassava leaves harvested at different time points post-inoculation using an RNeasy Plant Mini Kit (Qiagen, Hilden, Germany). RNA quality was assessed on an Agilent Bioanalyzer 2100 (Agilent Technologies, Santa Clara, CA, USA), and only samples with RNA integrity numbers (RIN) greater than 7 were used for library construction. Strand-specific RNA-seq libraries were constructed using a NEBNext^®^ Ultra™ II Directional RNA Library Prep Kit (NEB, Ipswich, MA, USA), following the manufacture’s protocol. After passing quality control (quantified by a Qubit^®^ 2.0 Fluorometer (Thermo Fisher Scientific, Waltham, MA, USA), size distribution of approximately 400 bp on the Agilent Bioanalyzer 2100), the libraries were subjected to paired-end sequencing (2 × 150 bp) on the Illumina NovaSeq 6000 platform. Three biological replicates were sequenced for each time point. Based on the sixty-six cassava–*X. phaseoli* pv. *manihotis* transcriptome datasets, the mean value (MV) of TPM (Transcripts Per Million) and standard deviation (SD) of TPM for each gene in each series were calculated. Furthermore, the coefficient of variation (CV, SD of TPM/mean TPM) was calculated. The genes were sorted from the smallest CV to highest CV. Genes with an average mean value (MV) greater than 50 and an average CV value less than 20% were defined as high expression levels and stable expression. Thirty-two candidate reference genes were selected (Table S4). Another seven reference genes were obtained from published literature. The gene-specific primers for all genes used for RT-qPCR were designed using the online tool Primer3plus (https://www.primer3plus.com/, accessed on 20 August 2025) with the given criteria: melting temperatures (Tm) between 55 and 65 °C, GC percentage between 45 and 60%, primer sequences spanning 18–25 base pairs, with amplicon sizes between 100 and 230 base pairs. The specificity of the primer pair sequences was further checked in Phytozome using the BLAST tool (https://blast.ncbi.nlm.nih.gov/Blast.cgi accessed on 20 August 2025) All primer sets were synthesized at Sangon Biotechnology Co. (Shanghai, China). Primer sequences are shown in Table S1.

### 4.4. RT-qPCR Analyses

RT-qPCR was conducted on a qTOWER^3^ Real-Time Thermal Cycler (Analytikjena, Jena, Germany) with 384-well PCR plates. Reaction mixtures consisted of 10 µL Hieff UNICON^®^ Universal Blue qPCR SYBR Green Master Mix (Yeasen, Shanghai, China), 0.4 µL of each 10 mM primer, ~100 ng cDNA template, with the remaining volume adjusted to 20 µL with sterile water. The thermal cycling conditions were as follows: 95 °C for 2 min, then 40 cycles of 15 s at 95 °C, and 34 s at 60 °C. After each amplification, a melting curve analysis was conducted between 60 °C and 95 °C to confirm product specificity. The RT-qPCR experiment was carried out with three biological replicates for each condition, and three technical replicates were set for each biological replicate. No template controls were included for each primer pair. A series of five-fold dilutions of the cDNA template was applied to establish the standard curves for calculating the amplification efficiency and correlation coefficients (R^2^) for every candidate reference gene [57].

### 4.5. Stability Assessment of Candidate Reference Genes

Box-plots of cycle threshold (Ct) values of the 39 candidate reference genes were plotted using GraphPad Prism 10 software (V10.4.0). Four statistical algorithms, geNorm v3.5. [37], NormFinder v20. [38], Delta Ct [39], and RefFinder [40] (http://blooge.cn/RefFinder/, accessed on 20 August 2025), were used to assess the reference gene expression stability across all five groups. Briefly, geNorm calculates a gene stability measure (M) based on the average pairwise variation between all candidate genes, NormFinder employs a model-based variance estimation approach, and the Delta-Ct method is a correlation-based approach that compares the relative pairwise expression levels between all pairs of candidate genes within each sample. For geNorm and NormFinder analysis, the original Ct value needs to be transformed into relative quantification data using the Delta Ct method. The GeNorm algorithm gives the stability value (M) and the pairwise variation value (V). The NormFinder algorithm calculates the stability value and standard error. The Delta CT method produces the mean standard deviation (mSD) value. Finally, the RefFinder website was used to comprehensively assess the stability of candidate reference genes by calculating the geomean of ranking values. BestKeeper software (V1.0) can only compare up to ten candidate reference genes together with ten target genes; therefore, BestKeeper was excluded from this study [58].

### 4.6. Validation of Reference Genes

The reliability of the screened reference genes was validated by examining the relative expression patterns of *MeNAC35* and *MeSWEET10a* genes in response to *Xpm* infection. Both the top-two stable and the bottom-two unstable reference genes calculated by the RefFinder algorithm were chosen for normalization. The relative expression level was determined by the 2^−∆∆CT^ approach using the raw data [57]. Three independent repetitions were performed on each sample.

## Figures and Tables

**Table 1:** Gene information, amplification length, efficiency, and R^2^ values.

Gene	Identifier	Description	Length (bp)	E (%)	R^2^	Notes
*MeSNRP70*	*Manes.01G134400*	U1 small nuclear ribonucleoprotein 70 kDa	106	99.7	0.996	This study
*MeTRA2*	*Manes.01G151200*	Transformer-2 protein	228	101.0	0.994	This study
*Me* *hn* *RNPR*	*Manes.01G240900*	Heterogeneous nuclear ribonucleoprotein R	171	104.6	0.998	This study
*MeDDX6*	*Manes.01G259600*	ATP-dependent RNA helicase DDX6	187	93.0	0.992	This study
*MeRBP* *1-* *1*	*Manes.01G274200*	Predicted RNA-binding protein	205	107.0	0.994	This study
*MePLAC8*	*Manes.02G019200*	PLAC8, cystine-rich-domain-containing protein	121	90.7	0.997	This study
*MePRPF38B*	*Manes.02G080000*	Pre-mRNA-splicing factor 38B family (PRPF38B)	227	107.0	0.995	This study
*Me14-3-3*	*Manes.02G151900*	14-3-3 protein	177	97.5	0.997	This study
*MeMBF1-1*	*Manes.03G100900*	Transcription factor MBF1	137	107.8	0.987	This study
*MeCWC15*	*Manes.03G143400*	Cell cycle control protein CWC15	182	105.1	0.987	This study
*MeMTAPase*	*Manes.04G078800*	MTA nucleosidase	133	97.3	0.996	This study
*MeCSNK1*	*Manes.04G141300*	Casein kinase 1-like protein 2	108	92.0	0.995	This study
*MeRBP1-2*	*Manes.05G055400*	Predicted RNA-binding protein	210	106.8	0.996	This study
*MeSKP1*	*Manes.05G144500*	SKP1-like protein	173	106.7	0.994	This study
*MeAL6*	*Manes.06G014800*	PHD finger protein Alfin-like 6	148	102.6	0.985	This study
*MeHisD*	*Manes.06G058300*	Histidinol dehydrogenase	135	100.0	0.982	[43]
*MeC3H43*	*Manes.06G114200*	Zinc Finger CCCH protein 43	194	93.8	0.987	[43]
*MeSRSF3*	*Manes.06G143500*	Serine-arginine rich splicing factor 3	150	102.7	0.997	This study
*MeSAP2*	*Manes.06G166800*	A20/AN1-containing stress-associated protein 2	210	108.0	0.996	This study
*MePABP2*	*Manes.07G073000*	Polyadenylate-binding protein 2	208	95.4	0.996	This study
*MeTUB*	*Manes.08G061700*	Tubulin-6	180	105.5	0.996	[44]
*MeHINT1*	*Manes.08G065600*	Histidine triad (HIT) family protein HINT1	167	107.5	0.997	This study
*MeBTF3*	*Manes.09G005100*	RNA polymerase II general transcription factor BTF3	192	106.3	0.998	[43]
*MeH1*	*Manes.09G031800*	Linker histone H1	173	91.0	0.996	This study
*MePP2A*	*Manes.09G039900*	Protein phosphatase 2A	150	90.9	0.989	[41]
*MeUBL5*	*Manes.09G068300*	Ubiquitin-like protein 5	154	107.5	0.989	This study
*MeRPL27*	*Manes.09G073000*	Large subunit ribosomal protein L27e	189	102.1	0.997	This study
*MeGTPb*	*Manes.09G086600*	GTP-binding family protein	184	96.8	0.998	[45]
*MeSCL30*	*Manes.10G029600*	Serine-arginine-rich SC35-like splicing factor SCL30	164	100.0	0.992	This study
*MeCHMP5*	*Manes.10G060100*	Charged multivesicular body protein 5	122	91.3	0.988	This study
*MeBDF1*	*Manes.10G093200*	Transcription initiation factor TFIID, subunit BDF1	179	108.5	0.998	This study
*MeRBX1*	*Manes.11G019000*	RING-box protein 1	210	98.3	0.999	This study
*MeBZW2*	*Manes.12G005200*	Basic leucine zipper and W2 protein	120	91.3	0.997	This study
*MeACT*	*Manes.13G086400*	Actin	149	106.6	0.999	This study
*MeEF1α*	*Manes.15G054800*	Elongation factor 1-alpha	139	104.9	0.993	[46]
*MeMBF1-2*	*Manes.15G095200*	Transcription factor MBF1	201	91.9	0.996	This study
*MeCDC25*	*Manes.16G055400*	Phosphatase CDC25	198	90.8	0.996	This study
*MeTIA1*	*Manes.16G093200*	Apoptosis-promoting RNA-binding protein TIA-1	210	109.0	0.999	This study
*MeEIF2*	*Manes.18G139800*	Translation initiation factor 2 subunit 2 (EIF2S2)	147	105.9	0.997	This study

**Table 2:** Stability of expression of 39 reference genes in *X. phaseoli* pv. *manihotis*-infected cassava leaves calculated by geNorm.

	All Samples		SC9		GR891		JG1301		154	
Gene	Rank	M	Rank	M	Rank	M	Rank	M	Rank	M
*MeSNRP70*	26	0.549	21	0.520	14	0.319	31	0.584	27	0.391
*MeTRA2*	10	0.395	12	0.429	1	0.222	9	0.310	1	0.187
*MehnRNPR*	1	0.290	1	0.256	13	0.311	1	0.199	9	0.261
*MeDDX6*	36	0.739	33	0.722	32	0.571	36	0.703	36	0.598
*MeRBP1-1*	6	0.349	7	0.367	10	0.295	7	0.288	16	0.298
*MePLAC8*	28	0.578	17	0.478	28	0.497	30	0.563	32	0.491
*MePRPF38B*	5	0.337	13	0.440	5	0.257	8	0.300	11	0.272
*Me14-3-3*	17	0.463	16	0.468	18	0.357	19	0.423	24	0.358
*MeMBF1-1*	16	0.455	20	0.507	3	0.236	18	0.411	18	0.311
*MeCWC15*	11	0.406	19	0.496	9	0.287	24	0.485	17	0.304
*MeMTAPase*	7	0.363	1	0.256	7	0.270	16	0.385	3	0.198
*MeCSNK1*	15	0.447	3	0.271	12	0.307	20	0.432	19	0.317
*MeRBP1-2*	1	0.290	4	0.307	8	0.278	4	0.259	8	0.253
*MeSKP1*	8	0.371	10	0.402	1	0.222	15	0.372	4	0.205
*MeAL6*	3	0.319	5	0.328	11	0.301	3	0.229	6	0.232
*MeHisD*	29	0.595	27	0.628	33	0.588	29	0.546	31	0.472
*MeC3H43*	35	0.717	25	0.592	31	0.552	35	0.675	35	0.567
*MeSRSF3*	14	0.437	8	0.379	16	0.335	14	0.359	25	0.369
*MeSAP2*	9	0.384	14	0.449	17	0.345	10	0.316	12	0.277
*MePABP2*	21	0.500	23	0.552	19	0.372	17	0.399	20	0.324
*MeTUB*	33	0.671	35	0.755	38	0.682	34	0.646	29	0.433
*MeHINT1*	38	0.807	39	0.877	29	0.514	38	0.771	38	0.673
*MeBTF3*	25	0.539	28	0.644	23	0.428	22	0.459	22	0.341
*MeH1*	20	0.490	24	0.573	25	0.452	1	0.199	14	0.287
*MePP2A*	34	0.694	38	0.832	37	0.653	33	0.624	34	0.535
*MeUBL5*	22	0.509	29	0.659	26	0.466	6	0.281	5	0.222
*MeRPL27*	18	0.472	18	0.486	21	0.402	12	0.335	15	0.293
*MeGTPb*	27	0.563	32	0.702	30	0.532	28	0.535	21	0.333
*MeSCL30*	24	0.529	31	0.688	27	0.480	11	0.325	13	0.282
*MeCHMP5*	37	0.761	34	0.739	36	0.636	37	0.731	37	0.631
*MeBDF1*	23	0.518	22	0.531	24	0.440	23	0.472	26	0.380
*MeRBX1*	4	0.331	11	0.417	4	0.248	5	0.273	10	0.266
*MeBZW2*	30	0.612	26	0.611	35	0.619	26	0.509	28	0.411
*MeACT*	31	0.631	36	0.776	20	0.388	25	0.496	30	0.453
*MeEF1α*	19	0.481	6	0.349	22	0.415	13	0.345	23	0.350
*MeMBF1-2*	32	0.649	30	0.673	34	0.604	32	0.605	33	0.514
*MeCDC25*	39	0.865	37	0.802	39	0.730	39	0.828	39	0.751
*MeTIA1*	12	0.417	15	0.458	15	0.327	21	0.445	7	0.242
*MeEIF2*	13	0.426	9	0.392	6	0.260	27	0.522	1	0.187

**Table 3:** Stability of expression of 39 reference genes in *X. phaseoli* pv. *manihotis*-infected cassava leaves calculated by NormFinder.

	All Samples		SC9		GR891		JG1301		154	
Gene	Rank	M	Rank	M	Rank	M	Rank	M	Rank	M
*MeSNRP70*	22	0.361	16	0.300	15	0.268	30	0.491	27	0.343
*MeTRA2*	19	0.348	4	0.233	14	0.253	15	0.288	19	0.271
*MehnRNPR*	1	0.186	6	0.245	1	0.147	1	0.168	1	0.137
*MeDDX6*	36	0.631	33	0.570	33	0.456	36	0.695	36	0.655
*MeRBP1-1*	9	0.284	19	0.370	19	0.294	8	0.241	13	0.233
*MePLAC8*	30	0.483	23	0.410	28	0.409	33	0.529	32	0.480
*MePRPF38B*	2	0.212	1	0.160	2	0.156	3	0.172	10	0.215
*Me14-3-3*	8	0.257	18	0.347	9	0.233	5	0.212	7	0.195
*MeMBF1-1*	4	0.237	8	0.259	6	0.223	4	0.199	4	0.178
*MeCWC15*	21	0.358	14	0.294	18	0.288	29	0.459	28	0.353
*MeMTAPase*	12	0.306	7	0.250	10	0.234	25	0.367	23	0.289
*MeCSNK1*	3	0.215	5	0.238	4	0.197	2	0.169	6	0.185
*MeRBP1-2*	6	0.238	10	0.262	7	0.225	10	0.246	11	0.226
*MeSKP1*	15	0.321	17	0.305	8	0.228	26	0.392	15	0.245
*MeAL6*	7	0.249	2	0.215	12	0.239	9	0.244	16	0.246
*MeHisD*	27	0.414	25	0.472	31	0.447	24	0.365	29	0.372
*MeC3H43*	33	0.596	20	0.380	29	0.410	34	0.643	35	0.625
*MeSRSF3*	14	0.320	21	0.381	17	0.287	16	0.295	21	0.280
*MeSAP2*	11	0.292	9	0.260	11	0.237	18	0.320	3	0.172
*MePABP2*	13	0.319	29	0.505	13	0.240	7	0.241	2	0.160
*MeTUB*	35	0.629	35	0.588	38	0.763	35	0.670	30	0.476
*MeHINT1*	38	1.056	39	1.103	34	0.499	38	0.936	38	0.906
*MeBTF3*	24	0.371	28	0.504	21	0.303	13	0.284	9	0.198
*MeH1*	23	0.367	27	0.497	24	0.362	6	0.227	14	0.238
*MePP2A*	34	0.620	38	0.862	37	0.571	31	0.512	33	0.494
*MeUBL5*	26	0.385	31	0.544	26	0.404	14	0.284	12	0.230
*MeRPL27*	16	0.328	22	0.394	22	0.322	20	0.321	18	0.265
*MeGTPb*	25	0.374	26	0.483	30	0.434	23	0.347	5	0.182
*MeSCL30*	28	0.415	30	0.543	25	0.371	11	0.270	24	0.291
*MeCHMP5*	37	0.667	34	0.580	36	0.537	37	0.745	37	0.752
*MeBDF1*	20	0.349	15	0.299	27	0.408	21	0.333	26	0.342
*MeRBX1*	5	0.238	3	0.217	5	0.199	12	0.274	8	0.197
*MeBZW2*	29	0.452	24	0.440	35	0.500	22	0.334	25	0.314
*MeACT*	32	0.535	36	0.667	23	0.342	27	0.394	34	0.528
*MeEF1α*	18	0.340	13	0.284	16	0.280	19	0.320	20	0.274
*MeMBF1-2*	31	0.511	32	0.550	32	0.451	32	0.514	31	0.478
*MeCDC25*	39	1.287	37	0.791	39	1.072	39	1.266	39	1.492
*MeTIA1*	10	0.291	11	0.276	20	0.298	17	0.305	17	0.248
*MeEIF2*	17	0.338	12	0.276	3	0.183	28	0.429	22	0.289

**Table 4:** Stability of expression of 39 reference genes in *X. phaseoli* pv. *manihotis*-infected cassava leaves calculated by Delta Ct.

	All Samples		SC9		GR891		JG1301		154	
Gene	Rank	M	Rank	M	Rank	M	Rank	M	Rank	M
*MeSNRP70*	25	0.810	17	0.770	15	0.620	27	0.720	31	0.950
*MeTRA2*	18	0.760	7	0.700	9	0.590	18	0.600	11	0.670
*MehnRNPR*	1	0.640	2	0.680	2	0.550	1	0.530	1	0.610
*MeDDX6*	36	1.110	33	1.030	33	0.850	36	1.110	36	1.170
*MeRBP1-1*	7	0.710	19	0.780	16	0.630	13	0.600	5	0.650
*MePLAC8*	29	0.910	22	0.830	28	0.800	31	0.870	30	0.940
*MePRPF38B*	2	0.650	1	0.670	1	0.540	4	0.560	2	0.610
*Me14-3-3*	9	0.710	18	0.770	13	0.610	20	0.610	12	0.670
*MeMBF1-1*	8	0.710	11	0.730	8	0.580	8	0.580	10	0.660
*MeCWC15*	20	0.770	15	0.750	14	0.620	25	0.680	29	0.840
*MeMTAPase*	11	0.720	5	0.690	10	0.590	22	0.630	23	0.770
*MeCSNK1*	6	0.690	4	0.680	5	0.570	9	0.580	8	0.660
*MeRBP1-2*	3	0.670	8	0.700	6	0.580	11	0.590	6	0.650
*MeSKP1*	12	0.720	14	0.740	7	0.580	7	0.570	22	0.770
*MeAL6*	5	0.670	3	0.680	11	0.590	12	0.590	3	0.640
*MeHisD*	28	0.870	25	0.920	31	0.840	29	0.790	26	0.800
*MeC3H43*	33	1.070	23	0.840	29	0.800	35	1.070	34	1.100
*MeSRSF3*	14	0.740	20	0.790	18	0.640	24	0.660	16	0.700
*MeSAP2*	10	0.720	12	0.730	12	0.610	3	0.550	15	0.690
*MePABP2*	19	0.760	27	0.940	17	0.630	6	0.570	14	0.680
*MeTUB*	34	1.100	35	1.050	38	1.220	30	0.850	35	1.110
*MeHINT1*	38	1.650	39	1.700	34	0.870	38	1.430	38	1.480
*MeBTF3*	24	0.810	29	0.950	21	0.680	15	0.600	20	0.730
*MeH1*	21	0.790	26	0.930	24	0.730	10	0.580	4	0.640
*MePP2A*	35	1.100	38	1.390	37	0.970	34	0.930	33	0.970
*MeUBL5*	23	0.810	30	0.980	27	0.770	5	0.560	9	0.660
*MeRPL27*	15	0.750	21	0.810	22	0.690	17	0.600	17	0.700
*MeGTPb*	27	0.840	28	0.950	30	0.830	16	0.600	25	0.790
*MeSCL30*	26	0.830	31	0.990	25	0.750	21	0.610	13	0.670
*MeCHMP5*	37	1.150	34	1.050	36	0.940	37	1.220	37	1.220
*MeBDF1*	22	0.790	16	0.770	26	0.750	26	0.710	21	0.760
*MeRBX1*	4	0.670	6	0.690	4	0.560	2	0.550	7	0.650
*MeBZW2*	30	0.910	24	0.900	35	0.890	28	0.740	24	0.770
*MeACT*	32	0.990	36	1.150	23	0.710	32	0.900	27	0.810
*MeEF1α*	17	0.760	10	0.730	20	0.660	23	0.640	18	0.710
*MeMBF1-2*	31	0.980	32	0.990	32	0.850	33	0.900	32	0.960
*MeCDC25*	39	1.940	37	1.290	39	1.620	39	2.200	39	1.890
*MeTIA1*	13	0.730	13	0.740	19	0.640	14	0.600	19	0.730
*MeEIF2*	16	0.760	9	0.720	3	0.550	19	0.610	28	0.840

**Table 5:** Stability of expression of 39 reference genes in *X. phaseoli* pv. *manihotis*-infected cassava leaves calculated by RefFinder.

	All Samples		SC9		GR891		JG1301		154	
Gene	Rank	M	Rank	M	Rank	M	Rank	M	Rank	M
*MeSNRP70*	25	20.76	9	8.80	18	16.41	33	26.84	31	28.39
*MeTRA2*	21	18.04	7	7.77	7	7.64	16	14.41	10	10.15
*MehnRNPR*	1	1.73	3	4.18	2	3.74	1	2.41	1	3.29
*MeDDX6*	37	36.25	34	32.49	24	19.32	35	28.92	36	36.00
*MeRBP1-1*	8	9.86	17	16.31	21	17.66	7	9.24	22	15.44
*MePLAC8*	29	25.73	23	21.92	34	28.49	31	24.93	27	22.27
*MePRPF38B*	2	2.51	2	2.74	1	3.56	2	4.28	4	6.03
*Me14-3-3*	9	9.95	20	17.66	12	12.05	6	8.22	23	16.10
*MeMBF1-1*	7	7.74	12	9.30	6	7.23	9	9.64	11	10.99
*MeCWC15*	22	19.61	15	12.75	17	16.05	36	29.40	28	23.58
*MeMTAPase*	13	12.79	4	4.79	11	10.88	27	23.99	18	14.20
*MeCSNK1*	6	6.34	5	5.18	8	8.71	4	6.32	13	11.66
*MeRBP1-2*	3	4.90	11	9.26	9	9.17	8	9.29	15	12.94
*MeSKP1*	15	13.34	16	14.62	4	5.55	26	23.24	7	9.48
*MeAL6*	5	5.96	1	2.51	14	14.91	5	6.45	12	11.28
*MeHisD*	19	17.21	27	25.98	28	21.71	13	11.60	32	28.41
*MeC3H43*	33	33.99	21	19.66	13	12.71	29	24.57	35	35.00
*MeSRSF3*	17	15.85	18	17.19	26	20.78	17	16.76	25	19.29
*MeSAP2*	12	11.58	13	10.84	15	15.54	18	16.87	3	6.00
*MePABP2*	11	11.17	30	28.18	10	10.59	10	10.00	2	4.68
*MeTUB*	35	34.48	35	33.39	38	38.00	37	34.75	14	12.71
*MeHINT1*	38	38.00	39	39.00	35	33.37	38	38.00	38	38.00
*MeBTF3*	10	10.95	29	27.94	23	19.06	11	10.54	19	14.28
*MeH1*	23	19.83	26	23.79	31	25.42	3	4.41	8	9.95
*MePP2A*	34	34.25	38	37.75	36	33.55	30	24.69	34	33.75
*MeUBL5*	28	24.64	32	30.67	32	26.74	14	11.73	6	7.40
*MeRPL27*	18	16.46	24	23.32	16	15.71	20	17.69	16	13.39
*MeGTPb*	26	22.47	28	26.13	27	21.56	23	18.32	17	13.87
*MeSCL30*	24	20.44	25	23.73	30	24.68	15	13.79	24	18.27
*MeCHMP5*	36	35.11	31	30.14	20	17.48	32	26.68	37	37.00
*MeBDF1*	27	24.04	19	17.36	33	27.71	25	23.09	30	27.39
*MeRBX1*	4	5.07	6	6.50	5	5.57	12	10.77	5	6.16
*MeBZW2*	32	28.97	22	21.40	37	34.49	22	18.20	29	23.66
*MeACT*	30	26.27	36	35.23	25	19.62	28	24.26	26	21.04
*MeEF1α*	14	13.06	14	11.45	19	16.68	19	17.27	20	14.34
*MeMBF1-2*	31	28.35	33	32.16	29	23.66	21	17.71	33	31.14
*MeCDC25*	39	39.00	37	37.25	39	39.00	39	39.00	39	39.00
*MeTIA1*	16	13.44	10	8.96	22	17.90	24	20.29	21	14.95
*MeEIF2*	20	17.41	8	8.74	3	4.82	34	28.91	9	10.01

## Data Availability

All data are contained within the article and Supplementary Materials.

## Supplementary Materials

The following supporting information can be downloaded at: https://www.mdpi.com/article/10.3390/plants14233655/s1, Figure S1: The melting curves of 39 candidate reference genes; Table S1: Primer sequence of all reference genes; Table S2: Ct values of the 39 candidate reference genes in four cassava varieties at six time points post-inoculation by *Xpm*; Table S3: Pairwise variation analysis of 39 candidate in five groups; Table S4: The TPM values of each candidate reference gene in different cassava genotypes under *Xpm* infection.
