# Supplementary material for: Transcriptome-Based Identification of Reference Genes for Expression Analysis in Cassava Under Xanthomonas phaseoli pv. manihotis Infection
Source: Plants (Basel). 2025 Nov 30;14(23):3655. doi: 10.3390/plants14233655 (PMC12693826; doi:10.3390/plants14233655)
Supplement: Supplementary file 1 [file plants-14-03655-s001.zip › Table S1 Primer sequence of all reference genes.pdf]

Table S1 Primer sequence of all reference genes

No.	Identifier	Forward Primer (5'-3')	Reverse Primer (5'-3')
1	*Manes.01G134400*	TGGGTGATTACAACGATGCC	TGCCCAATCAGTTTGAGCTG
2	*Manes.01G151200*	TGGCAGATCAAGATCCAGAAGC	ACGCATCGGTTTGCATCATC
3	*Manes.01G240900*	GGCCAAGTGTTGGAAGTTGT	ACCAAATCCAGAACCAGCAC
4	*Manes.01G259600*	GATGTTGCAAGATCACCGCA	TCCAAACCTCCCTGATCGAC
5	*Manes.01G274200*	TTTGTTGGGTGACGATGACG	ACGGCCAGCTTCATTCTTTG
6	*Manes.02G019200*	ACGCATTGATCAGCCCTTTC	AGGTGGTGGGTAACTGGATG
7	*Manes.02G080000*	AACGGGAAAGAGATCGGTACAG	TTGCAAACTTCGGCTTCTGC
8	*Manes.02G151900*	TCGCAAAGAAGCTGCAGATC	AATGCTTGTTTGGCCAGGTG
9	*Manes.03G100900*	GGCTCGAATGGACAAGAAAC	CAAGAGCCCTCTCCAGTTTG
10	*Manes.03G143400*	CAAGACACCCAGGATGAATTGC	ACACTGCGTGGAATAATGCG
11	*Manes.04G078800*	AGGCCCAATTTCCAATATCC	ACCATGATAGCGAACCCAAG
12	*Manes.04G141300*	TCGTGATGCAGCACTTGTTG	ACCATGGCCGAACTTCTTTG
13	*Manes.05G055400*	TTGTTGGGTGACGATGATGC	ACGGCCAGCTTCATTCTTTG
14	*Manes.05G144500*	AGCATCGCCGGCAAAATTAG	TGCTGGTGACATTAGGAAGAGG
15	*Manes.06G014800*	AATGCAATCTCGGCAACCTG	ATCAGTGCCATAGCTTTCGC
16	*Manes.06G058300*	GCTCGTGATATGGTTGAGGC	CGTCCATTGGCCCAAGAAAA
17	*Manes.06G114200*	ATCAGCAGCAGACACCAATG	ACAAATGTGCTGACCAGGTC
18	*Manes.06G143500*	GAGCAAGTTGACGGATCGAG	CTACGGTAAGGACTTGGCGA
19	*Manes.06G166800*	TCAAGCAACATGGAGCAAAC	AAAAAGGTTGCCACATCGAC
20	*Manes.07G073000*	ATGCCAATGCGTGATACAGC	ACTCCAGCAAGTGCAAAACC
21	*Manes.08G061700*	GTGGAGGAACTGGTTCTGGA	TGCACTCATCTGCATTCTCC
22	*Manes.08G065600*	ACTGGGCTGTCTAAGGCTGA	AGGAGGTGGATGTGAAGGTG
23	*Manes.09G005100*	TTTTGAAGCTGCTGCAGAGG	TGAAGCGGATCTGAACAACC
24	*Manes.09G031800*	TAAGAGGAGAGGGAGGCCAC	GAACTGGGGACTTCTTGGGG
25	*Manes.09G039900*	TGCAAGGCTCACACTTTCATC	CTGAGCGTAAAGCAGGGAAG
26	*Manes.09G068300*	GACACCATCGGAGACCTGAA	TGTAATAGAGCTCGAGGCCC
27	*Manes.09G073000*	AAGAAGTCCCGCGTTAAGTG	ATTCTTGCCCGTCTTGAACC
28	*Manes.09G086600*	GGTGGGTAACAAGGCAGACA	TGAGTCGGTATCTGCAAGCC
29	*Manes.10G029600*	TTCCCCAGGTAGAAGGCATG	CTCTTGCTTCCTCGGACTCA
30	*Manes.10G060100*	CACTGGAAGCAGACATGGGA	TGCCTGTCCTATTGGTGCTG
31	*Manes.10G093200*	AGAACAGATCCGGCAGTTGA	ATCGCTTCATGTCTTTGGCC
32	*Manes.11G019000*	ATGGACACTGACGTCACCATG	ACTTGCTTGATTCGCTTGGC
33	*Manes.12G005200*	ACCTGGCACAACAAAACCTG	CTGCGGCGCAAAATTTTCTG
34	*Manes.13G086400*	TTCGTGTCAAGGTGTCGTGA	GCCCTCTCATTTGCTGCAAT
35	*Manes.15G054800*	TGAACCACCCTGGTCAGATTGGAA	AACTTGGGCTCCTTCTCAAGCTCT
36	*Manes.15G095200*	ATGAGCGAGTGCCAAGTGAA	ACTTCTTCCCTCGCAGCTTC
37	*Manes.16G055400*	TGTCAAACGTCGCCCCAATA	GCACAAGTTGGACCACGAAC
38	*Manes.16G093200*	AATGGGAAGTGGCTCGGAAG	AACCTCAGGAGCCAGATTGC
39	*Manes.18G139800*	TTTGCGGGAGAACAATCCAG	ACATGATCTGGTTGCCGATG
MeNAC35	*Manes.03G114200*	AGGCCACTGGAACCGATAAA	TCCCCATTTTGCGAGAGGAT
*MeSWEET10*a	*Manes.06G123400*	GGTTCCACAGCCCATAGAC	GCACCACCACTTTGAACGAT
