# Supplementary figures and images for: Transcriptome-Based Identification of Reference Genes for Expression Analysis in Cassava Under Xanthomonas phaseoli pv. manihotis Infection
Source: Plants (Basel). 2025 Nov 30;14(23):3655. doi: 10.3390/plants14233655 (PMC12693826; doi:10.3390/plants14233655)

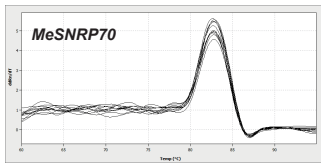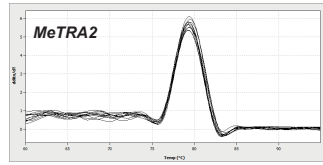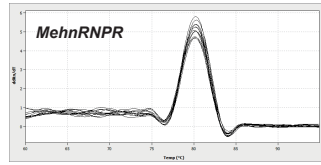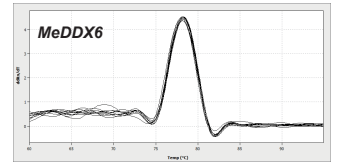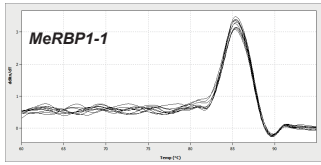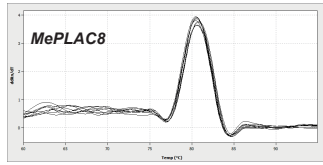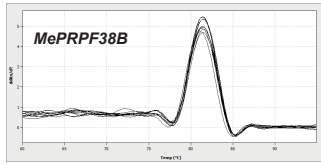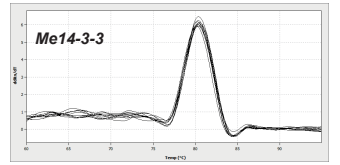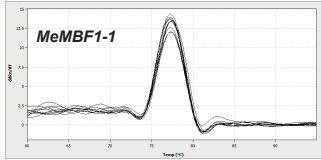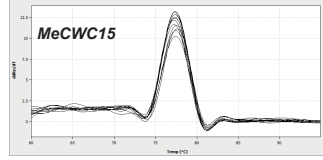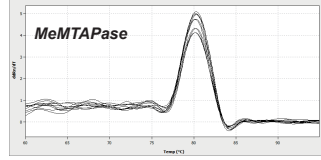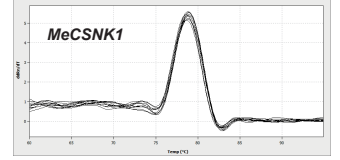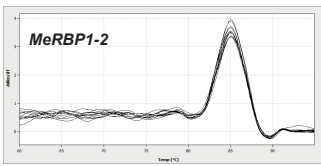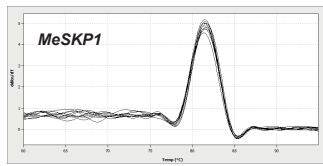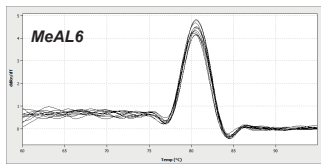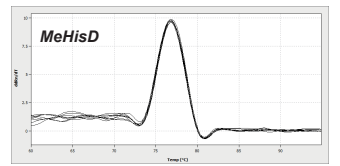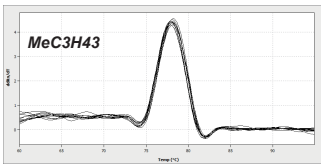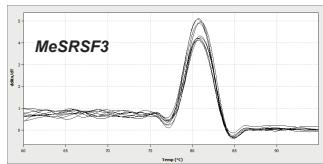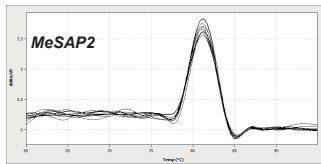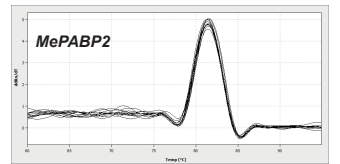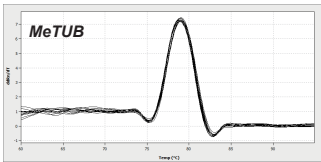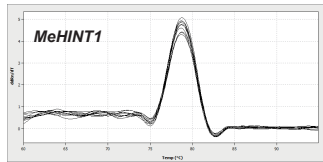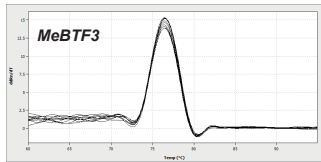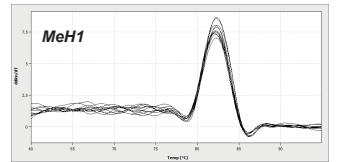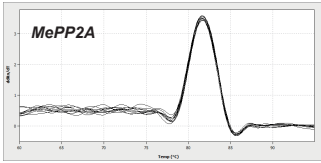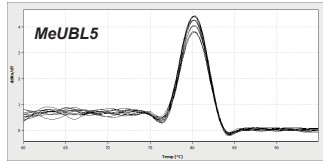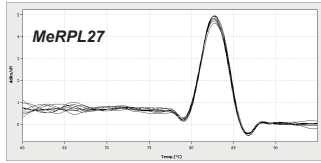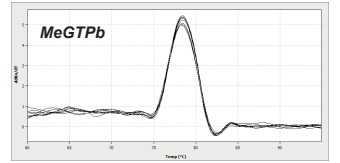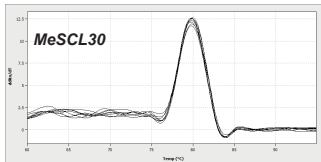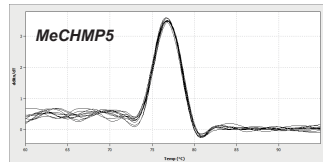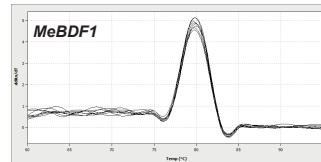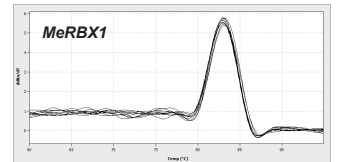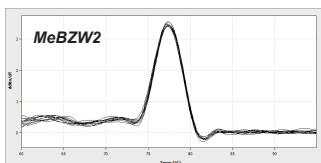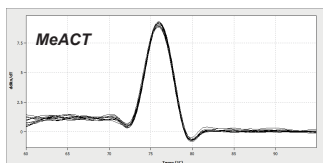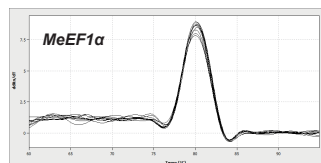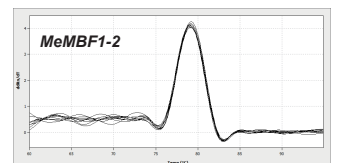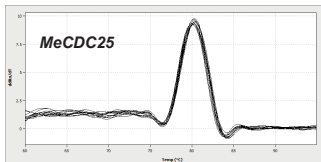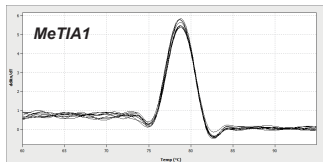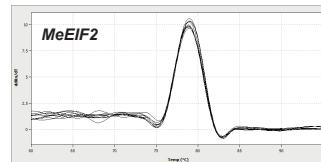

Supplement: Supplementary file 1 [file plants-14-03655-s001.zip › Figure S1 The melting curves of 39 candidate reference genes.pdf]
